# Microbiome alterations are related to an imbalance of immune response and bacterial translocation in BDL-rats

**DOI:** 10.22038/IJBMS.2019.36487.8753

**Published:** 2020-02

**Authors:** Natali Vega-Magaña, Antonio Galiana, Luis Felipe Jave-Suárez, Leonel Garcia-Benavides, Susana del Toro-Arreola, Jaime Federico Andrade-Villanueva, Luz Alicia González-Hernández, Rosa Cremades, Adriana Aguilar-Lemarroy, María Guadalupe Flores-Miramontes, Jesse Haramati, Jesús Meza-Arroyo, Miriam Ruth Bueno-Topete

**Affiliations:** 1 Instituto de Investigación en Enfermedades Crónico Degenerativas, Departamento de Biología Molecular y Genómica. Centro Universitario de Ciencias de la Salud, Universidad de Guadalajara. CP 44340, Guadalajara, Jalisco, México; 2 Instituto de Investigación en Inmunodeficiencias y VIH. Departamento de Clínicas Médicas. Centro Universitario de Ciencias de la Salud, Universidad de Guadalajara. CP 44340, Guadalajara, Jalisco, México; 3 FISABIO Fundación para el fomento de la Investigación Sanitaria y Biomédica de la comunidad de Valencia. CP 46015, España; 4 División de Inmunología, Centro de Investigación Biomédica de Occidente, Instituto Mexicano del Seguro Social. CP 44340, Guadalajara, Jalisco, México; 5 Departamento de Ciencias Biomédicas, Centro Universitario de Tonalá, Universidad de Guadalajara. CP 45425, Tonalá, Jalisco, México; 6 Unidad de VIH, Antiguo Hospital Civil de Guadalajara “Fray Antonio Alcalde”. CP 44280, Guadalajara, Jalisco, México; 7 Departamento de Microbiología y Patología, Centro Universitario de Ciencias de la Salud, Universidad de Guadalajara. CP 44340, Guadalajara, Jalisco, México; 8 Laboratorio de Inmunología, Departamento de Biología Celular y Molecular, Centro Universitario de Ciencias Biológicas y Agropecuarias, Universidad de Guadalajara. CP 44600, Guadalajara, Jalisco, México; 9 Departamento de Ciencias de la Salud, Centro Universitario de los Valles, Universidad de Guadalajara. CP 46600, Ameca, Jalisco, México

**Keywords:** Bile duct ligation, Colon, Dysbiosis, Immune response, Mesenteric lymph nodes, Pyrosequencing

## Abstract

**Objective(s):**

Bacterial translocation in patients with cirrhosis is an important triggering factor for infections and mortality. In the bile duct ligation (BDL) model, crucial players of bacterial translocation are still unknown. This study aims to determine the interrelation between microbiome composition in the colon, mesenteric lymph nodes, and liver, as well as the local inflammatory microenvironment in the BDL model.

**Materials and Methods:**

Liver damage was assayed by Masson trichrome staining, and hepatic enzymes. The diversity of microbiota in colon stools, mesenteric lymph nodes, and liver was determined by 16S rRNA pyrosequencing. Cytokine expression in mesenteric lymph nodes was analyzed by qRT-PCR.

**Results:**

Our results show that Proteobacteria was the predominant phylum found to translocate to mesenteric lymph nodes and liver in cirrhotic rats. Bile duct ligation induces a drastic intestinal dysbiosis, revealed by an increased relative abundance of *Sarcina, Clostridium, Helicobacter, Turicibacter*, and *Streptococcus* genera. However, beneficial bacteria, such as *Lactobacillus, Prevotella* and *Ruminococcus* were found to be notably decreased in BDL groups. Mesenteric pro-inflammatory (TNF-α, IL-1β, IL-6, TLR-4) and regulatory (TGF-β, Foxp3, and IL-10) molecules at 30 days post-BDL were significantly increased. Conversely, TGF-β and Foxp3 were significantly augmented at 8 days post-BDL.

**Conclusion:**

Dysbiosis in the colon and mesenteric lymph nodes is linked to an imbalance in the immune response; therefore, this may be an important trigger for bacterial translocation in the BDL model.

## Introduction

Innate and adaptive immune dysfunction play a pivotal role in the pathogenesis of liver disease (1). Bacterial translocation (BT) in chronic liver diseases is an important triggering factor of infections and sepsis. Bacterial translocation is defined as the migration of bacteria or bacterial products, such as lipopolysaccharide or DNA, from the intestinal lumen to mesenteric lymph nodes (MLNs) or extra-intestinal organs (2). Moreover, bacterial infections are responsible for 30% to 50% of deaths in cirrhosis. The most common pathogens involved in this disease are *Escherichia coli*, *Klebsiella spp*, and *Enterococcus faecalis*. Systemic alterations to the immune system in relation to BT have been thoroughly reported in the literature; the most notable of these include a decreased phagocytic capacity, lower production of complement molecules, and chronic inflammation (3). However, alterations of the inflammatory and regulatory immune responses in MLN that promote BT to the liver have yet to be well explored.

In patients with liver disease, it has been postulated that intestinal dysbiosis can promote BT and the progression of liver damage. A common feature of cirrhosis is an increase of potentially pathogenic bacteria (*Enterobacteriaceae *and *Streptococcus*), accompanied by a reduced proportion of beneficial bacteria (*Lachnospiriaceae *and Bifidobacteria)*.* Furthermore, in the CCl_4_ model of cirrhosis, high levels of Enterobacteria in MLNs have been associated with bacterial translocation (4). However, in the BDL model, De Minicis *et al.* did not find differences in cecum microbiota between sham and BDL groups (5). Notably, sequencing analysis of neither the colonic microbiome, nor, more significantly, that of the MLN and liver, has previously been reported in the experimental model of BDL. 

MLNs are a pivotal part of the immune system. They contain specialized cells that trigger immune responses and play a crucial role in the interplay with intestinal microbiota. Consequently, it is likely that the immunogenicity, and mechanisms used to evade the immune response, of these microorganisms dictate BT. Therefore, the aim of this study was to determine the implications of changes in the intestinal microbiome, and immune responses, in the permissibility of bacterial translocation in an experimental model of cholestasis.

## Materials and Methods

***Cholestasis induced by bile duct ligation***

Male Wistar rats weighing 200-250 g, were obtained from Charles River (Boston, MA, USA). Cholestasis was induced by bile duct ligation (BDL) according to the method of Lee *et al*, 1988 (6). Ligated animals (n=17) were divided into two groups, one corresponding to 8 days and the other one to 30 days of BDL evolution. Rats were housed depending of the group of study. At the conclusion of the experiment, 5 animals remained in control and BDL groups. Animals were sacrificed and subsequently the liver and MLN were obtained. This project was approved by the Ethics Committee of the University of Guadalajara based on technical specifications for the production, care, and use of laboratory animals, NOM-062-ZOO-1999, and the “Guide for the Care and Use of Laboratory Animals” prepared by the National Academy of Sciences and published by the National Institutes of Health in 1985.

***Histological analysis***

Representative hepatic samples from four lobes were taken and 4 % formalin fixed. Liver sections (5-μm thick) were stained with Masson’s trichrome. The amount of fibrosis was assessed based on the Ishak score (7).

***Biochemical analysis***

Serum hepatic enzymes including total bilirubin, alanine aminotransferase (ALT) and alkaline phosphatase were determined by an automated method.

***Pyrosequencing 454-GSjunior***

MLN and liver were removed under aseptic conditions. All the samples were immersed in sterile water and 70 % ethanol. Subsequently, the tissues were ground in 15 ml of Trypticase Soy broth (Becton Dickinson, Germany). Genomic DNA extraction was performed using the QIAamp^Ò^ DNA Mini Kit (Qiagen, Germany) according to the manufacturer’s instructions. Fecal samples were taken from the colon and immediately stored at -80 ^°^C. DNA isolation from colonic contents was made from 100 mg of stool, following the manufacturers instructions using the ZR Fecal DNA MiniPrep™ kit (Zymo Research, USA). The bacterial 16S rRNA gene was amplified by PCR in each sample in order to synthesize the amplicon libraries prior to the pyrosequencing of each sample, using forward (5’-TCCTACGGGAGGCAGCAGT-3’) and reverse (5’-GGACTACCAGGGTATCTAATCCTGTT-3’) primers for the amplification of the V3-V4 16S rRNA 466 bp size region. The amplification protocol used was 95 ^°^C -5 Minutes followed by 30 cycles of: 95 ^°^C, 30 sec, 60 ^°^C, 30 sec, 72 ^°^C, 20 sec, each sample was assigned a unique multiplex identifier (MID) and high fidelity Extensor Long Range PCR Enzyme (Thermo Fisher Scientific, USA) was used. 

Prior to pyrosequencing, 16S rRNA library amplicons were purified using the MinElute PCR purification kit (Qiagen, Germany) and then using Agencourt AMPure XP beads (Beckman Coulter, USA). The amplicon libraries were then pyrosequenced using the GS Junior Sequencer utilizing the GS Junior Titanium Sequencing Kit (Roche, Switzerland) and the GS Junior Titanium PicoTiter Plate Kit (Roche, Switzerland), following the manufacturer’s instructions. 

***Bioinformatics analysis and OTU assignment***

Raw sequences were analyzed using QIIME v 1.9.0 software (8). Chimeric sequences were eliminated and of the remaining sequences, only those measuring between 250 bp and 450 bp, with an end-trimming quality greater than 25 analyzed in windows of 50 bases, were used for taxonomic classification. These high quality partial sequences of the 16S rRNA gene were classified in Operational Taxonomic Units (OTUs) using the RDP database (Ribosomal Database Project) for the 16S rRNA gene with a bootstrap cut-off of 80% and only the OTUs representing over 0.1 % of the total sequences of each sample were considered in subsequent statistical analyses.

Calculation of a-diversity estimators as rarefaction curves and microbial diversity estimators such as Shannon-Wiener and Chao1 were performed using QIIME. The sequence dataset was divided into six subsets of samples: MLN of Sham, MLN of 8 days post-BDL, liver of 8 days post-BDL, and colon contents of Sham, 8 or 30 days post-BDL. This software was also used for b-diversity analysis by means of a principal coordinates analysis (PCoA) following a weighted unifrac algorithm. 

***qRT-PCR***

RNA was isolated from 100 mg of MLN using TRIzol reagent (Invitrogen, Belgium) and reverse transcribed. PCR conditions were 95 ^°^C (10 min) followed by 35 cycles of 95 ^°^C (15 sec), with specific annealing and extension temperatures and primer sequences as shown in Table 1. Conditions for β2-microglobulin, TGF-β1 and TNFα- were adapted from previous publications (9-11), whereas primers for IL-1β, IL-6, TLR4, Foxp3 and IL-10 were designed using the primer blast tool, and mRNA sequences from the NCBI data base. Gene expression analysis was performed by the 2^-DDCT^ method.

***Statistical analysis***

Data were analyzed using the SPSS 10.0 statistical package (SPSS Inc,). Results were expressed as mean±SD. Comparisons were performed using one-way ANOVA, followed by Bonferroni’s *Post hoc* test. *P*-values ≤0.05 were considered as statistically significant.

## Results

***Liver damage in BDL rats***

**Figure 1:**
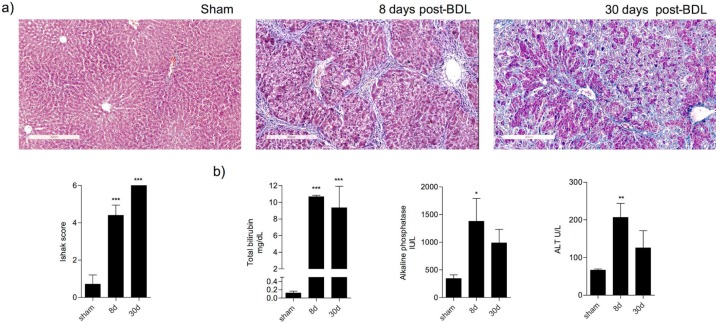
Liver damage in Bile Duct Ligation model

In order to evaluate liver fibrosis, the tissues were stained with Masson’s trichrome stain. In sham animals, hepatic sections exhibited a normal morphology of the portal triad and central vein. In BDL rats, extracellular matrix deposits and ductal proliferation were evident at 8 days of BDL. At 30 days post-BDL, fibrosis is clearly observed throughout the whole parenchyma. The Ishak analysis showed that the liver damage was gradually increased from 8 (*P<*0.001) to 30 days (*P<*0.001) post-BDL (Figure 1a). Biomarkers of liver damage increased significantly: total bilirubin (*P<*0.001), alkaline phosphatase (*P<*0.05), and alanine aminotransferase (*P<*0.01) increased in BDL rats in contrast with the sham group (Figure 1b).

***Bacterial translocation to mesenteric lymph nodes and liver***

**Figure 2:**
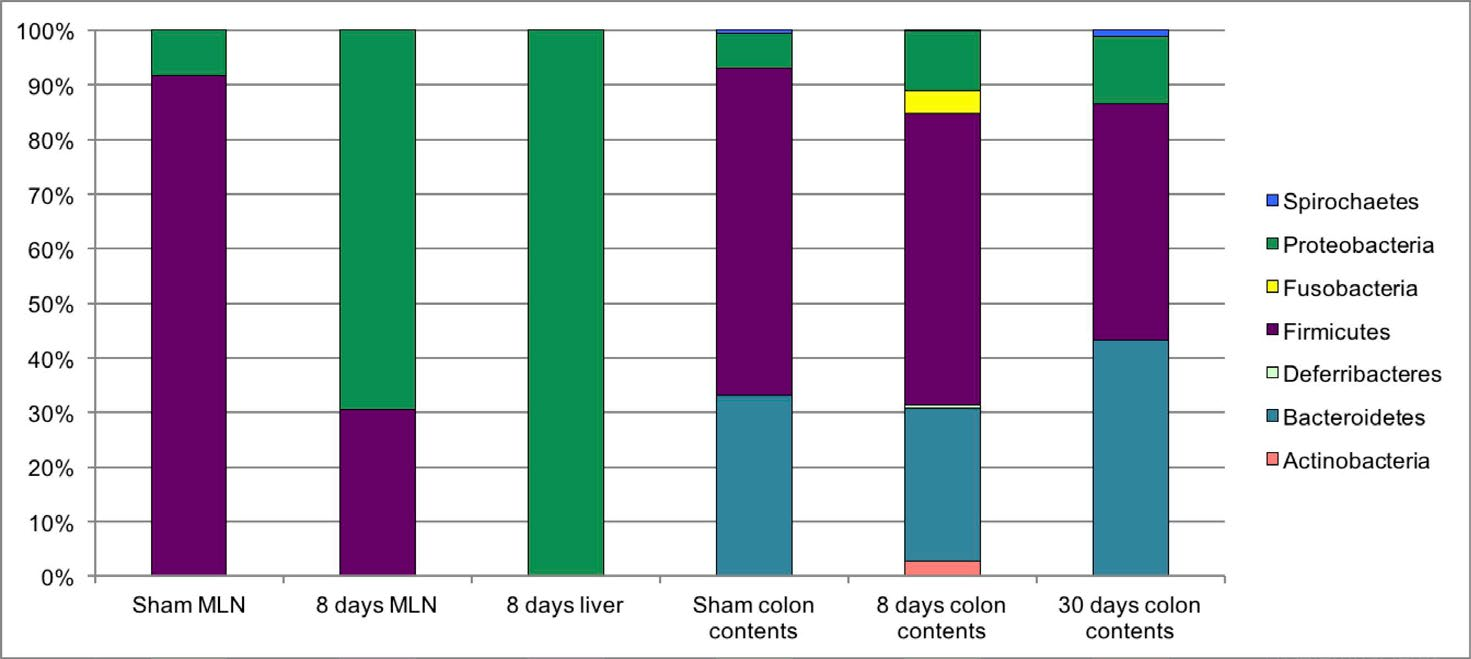
Microbiome phyla composition in mesenteric lymph nodes, liver and colon contents in BDL model. Relative abundance determined by 16S rRNA pyrosequencing GS-Junior 454. Shown data of sham MLN, eight days post-BDL MLN, eight days post-BDL liver, as well as colon contents of Sham, eight days post-BDL, and thirty days post-BDL groups. Sequences were analyzed using the QIIME (Quantitative Insights into Microbial Ecology) pipeline. Five rats per group MLN: mesenteric lymph nodes; BDL: bile duct ligation

Bacterial translocation can occur in normal physiological conditions. In the context of liver disease, bacterial translocation is an important trigger of inflammation and liver damage. To assess this variable, we performed 16S rRNA sequencing of the MLN and liver homogenates. We observed that rats with 8 days of liver damage via BDL were positive for 16S rRNA qPCR amplification in both organs. However, the sham control rats were positive in the MLN, but negative in the liver homogenates. The analysis of relative abundances of sequences revealed that the MLN of sham control rats showed 90.8 % Firmicutes and 8.08 % Proteobacteria. In contrast, the bacterial composition in the MLN of cirrhotic rats was found to be 30.18 % Firmicutes and 68.52% Proteobacteria. Liver homogenates exhibited mostly Proteobacteria (97.42%), and a minor proportion of Firmicutes (0.10%) (Figure 2).

***Bile duct ligation induces changes in colonic microbiota***

Intestinal bacterial composition is fundamental to the maintenance and stability of the mucosal barrier and the avoidance of bacteria or bacterial product leakage. As has been previously described, in liver fibrosis there persists an intestinal dysbiosis and small intestinal bacterial overgrowth, which in turn may lead to bacterial translocation and the perpetuation of liver damage. The goal of the present work was to determine the colonic bacterial diversity, in an experimental model of bile duct ligation, by using 16S rRNA sequencing. Our results showed that BDL induces a dramatic alteration in bacterial communities. The 8 days post-BDL group was found to have an increased abundance of Actinobacteria (2.66%) and Fusobacteria (3.88%) in comparison with the control group (0%). On the other hand, the abundance of Firmicutes notably decreased at 30 days post-BDL (39.0%), with respect to the control group (52.21%). In contrast, Bacteroidetes (38.92 %) and Proteobacteria (11.04%) increased in comparison to the sham group (28.93% and 5.70%, respectively) (Figure 2).

**Figure 3.**
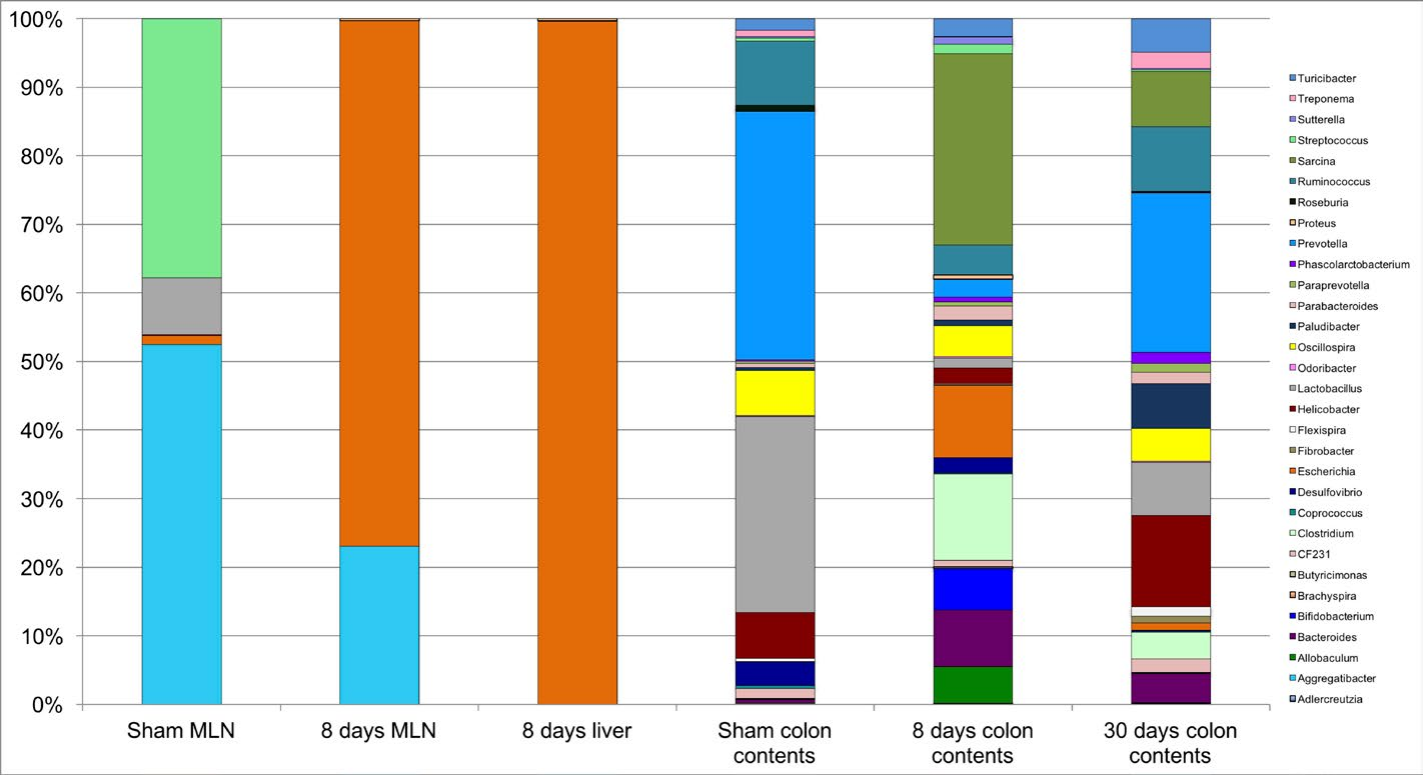
Microbiome genera composition in mesenteric lymph nodes, liver and colon contents in BDL model. Relative abundance determined by 16S rRNA pyrosequencing GS-Junior 454. Shown data of sham MLN, eight days post-BDL MLN, eight days post-BDL liver, as well as colon contents of Sham, eight days post-BDL, and thirty days post-BDL groups. Sequences were analyzed using the QIIME (Quantitative Insights into Microbial Ecology) pipeline. Five rats per group MLN: mesenteric lymph nodes; BDL: bile duct ligation

Subsequently, at the genus level we found an increased abundance of *Escherichia* in MLN and liver of BDL rats, in comparison to sham rats (Figure 3). In the colon contents, we observed an increased abundance of *Sarcina* (12.31%), *Clostridium* (5.57%), *Turicibacter* (1.14%), and *Streptococcus* (0.58%) at 8 days post-BDL, in contrast to the control group. Conversely, beneficial bacteria such as *Lactobacillus* (0.63%), *Prevotella* (1.1 %), and *Ruminococcus* (1.9%) were strongly decreased at 8 days post-BDL. Similarly, the 30 days post-BDL group demonstrated a decrease in the relative abundance of *Lactobacillus* (3.29%), and *Prevotella* (9.87%) with respect to the control group, while *Helicobacter* (5.62%), *Sarcina* (3.42 %), *Turicibacter* (2.07 %), and *Clostridium* (1.64 %) increased at 30 days post liver damage by BDL (Figure 3). 

***Alpha and beta diversity in MLN, liver and colonic contents***

**Figure 4:**
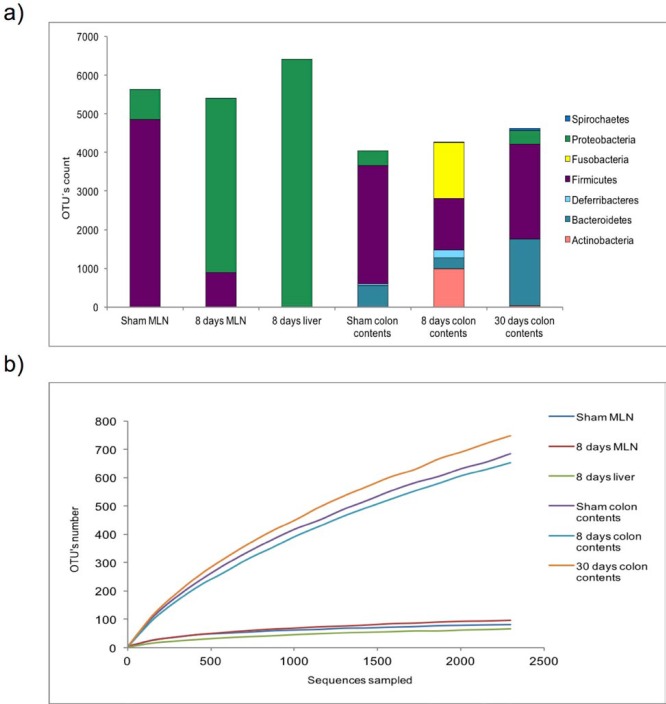
OTU counts and rarefaction plot. a) OTU counts of sham MLN, eight days post-BDL MLN, eight days post-BDL liver, as well as colon contents of Sham, eight days post-BDL, and thirty days post-BDL groups. b) rarefaction plot of sequences. Sequences were analyzed using the QIIME (Quantitative Insights into Microbial Ecology) pipeline. Five rats per group. OTU: operational taxonomic unit; MLN: mesenteric lymph nodes; BDL: bile duct ligation

In order to determine alpha diversity, we performed the Chao and Shannon indices. Our results showed a subtle increase in bacterial diversity in the MLN of cirrhotic rats (Chao1: 9.86; Shannon: 2.43), in comparison to the sham control group (Chao1: 9.28; Shannon: 2.20). Meanwhile, sequences from the 8 days post-BDL group liver presented less diversity (Chao1: 4.66; Shannon: 1.58). Colon content samples were found to be highly diverse, in contrast to organ samples. Nonetheless, the samples taken at 8 days post-BDL (Chao1: 36.3) exhibited a decreased colonic bacterial diversity, with respect to the sham control and 30 days post-BDL groups (Chao1: 46.5 and Chao1: 48.65, respectively) (Table 2). OTU counts and rarefaction plots are shown in Figure 4.

**Figure 5:**
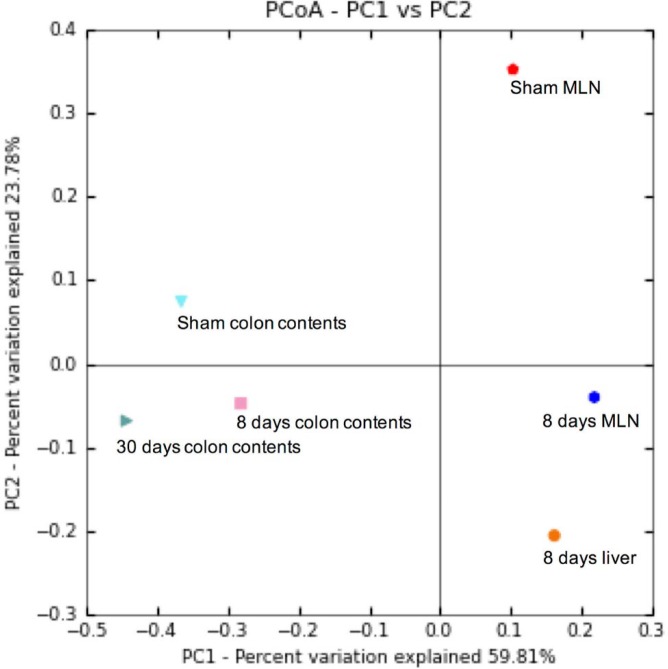
Principal coordinates analysis of weighted UniFrac distances. The graphic shows the phylogenic distances of groups: sham MLN, eight days post-BDL MLN, 8 days post-BDL liver, as well as colon contents of Sham, 8 days post-BDL, and 30 days post-BDL groups. Sequences were analyzed using the QIIME (Quantitative Insights into Microbial Ecology) pipeline. 5 rats per group. MLN: mesenteric lymph nodes; BDL: bile duct ligation

Subsequently, we used the UPGMA method based on the weighted UniFrac metric to compare groups. This analysis supports the conclusion that the MLN of rats with liver damage and those of the sham control group were composed of different bacterial communities. Furthermore, we also observed that the colonic microbial communities of the sham control group as well as the 8 and 30 days post-BDL groups are quite distinct (Figure 5).

***Bacterial translocation induces activation of immune responses***

**Figure 6:**
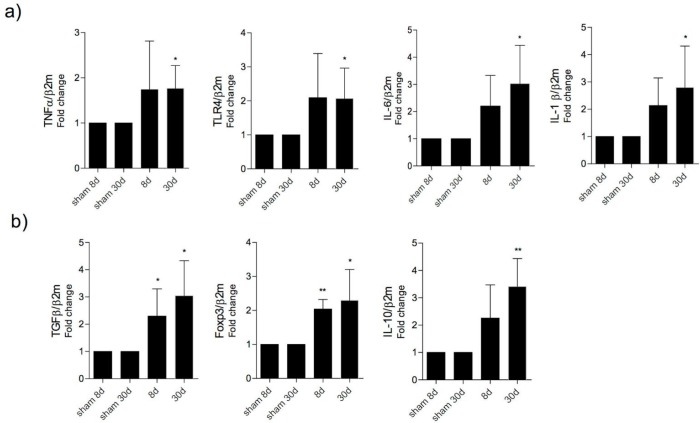
Gene expression of pro-inflammatory and regulatory molecules in mesenteric lymph nodes

To elucidate if the immune response had a role in containing bacterial translocation we determined pro and anti-inflammatory cytokines in the MLN by qRT-PCR. Our results showed an increased gene expression of *TNF-α (P<*0.05), *IL-1*β (*P<*0.05), *IL-6 *(*P<*0.05), and *TLR4* (*P<*0.05) at 30 days post-BDL, in comparison to the control group (Figure 6a). On the other hand, *TGF-*β* and Foxp3 *were significantly increased at 8 (*P<*0.05, *P<*0.01, respectively) and 30 days (*P<*0.05) post-BDL, when compared with the control group. Nevertheless, was an increasing tendency of pro-inflammatory cytokines at 8 days post-BDL. Moreover, *IL-10 *did show a significant increase only at 30 days post-BDL (*P<*0.01) (Figure 6b).

## Discussion

Bacterial translocation has been postulated as a transcendental mechanism in the pathogenesis of spontaneous bacterial infections in liver cirrhosis. Bacterial overgrowth is one of the main triggers of bacterial translocation. Bacterial translocation has been described in cirrhotic patients and in experimental models of chronic liver damage. While bacterial translocation is commonly associated with advanced stages of damage and hemodynamic alterations (12,13), Fouts *et al.*, 2012, demonstrated, using the BDL model in mice, that bacterial translocation occurs 24 hours after ligature (14). Therefore, we consider that this experimental model is very valuable for the understanding of the various mechanisms related to bacterial translocation from the early stages of the disease to its evolution to cirrhosis and is thus a useful model for our experiments described in this paper.

Our results show that bacterial translocation to the MLN and liver was evident at 8 days of liver damage by BDL. Interestingly, we observed that the sham operated group presented positive bacterial loads in the MLN, but were negative in the liver samples. Our data coincide with those of Ogata *et al.*, 2003, who also reported positivity in bacteriological cultures of the MLN of sham rats in the BDL model (15). A possible trigger for bacterial translocation is surgical stress (16), which may have influenced our results.

With respect to the bacterial diversity found in the MLN of control and BDL rats, we can infer that, in fact, a fundamental component in the containment of bacteria in mesenteric tissue is the immunogenicity of the bacteria and the ability of the immune system to limit the bacterial translocation to other organs. The results obtained in this work show that the bacterial composition in the MLN of the control group are mostly defined by organisms belonging to the Firmicutes phylum. In contrast, in the BDL group, bacteria of the Proteobacteria phylum predominate. These data agree with what has been previously reported by our research group, where we detected (using MALDI-TOF of isolated bacterial colonies) the presence of *E. coli* in the MLN of rats with BDL, while the sham control group was positive for *Staphylococcus* *chromogenes *(17).

When measuring the gene expression of molecules related to the regulation of the immune system and inflammation, we observed that during the early stage of liver damage (8 days post-BDL) only regulatory molecules (TGF-β and* Foxp3*) were significantly increased. Nonetheless, also we observed a tendency for an increase in the expression of genes of pro-inflammatory cytokines; these results were not statistically significant, possibly due to the limited sample size. We infer that the increase in regulatory molecules may be a mechanism to counteract the inflammatory process induced by the insult of translocated bacteria to the MLN. It is possible that by the timepoint that the experiments were performed, TGF-β and Foxp3 gene products had already been produced in significant numbers and had already mediated their down modulatory effects on the production of pro-inflammatory cytokines. Despite the limited increased in pro-inflammatory gene activity, the immune system was evidently not efficient in delimiting the passage of bacteria to the liver. Our results coincide with those of Yu *et al.*, 2014, where they reported an increase in regulatory T cells in the MLN at an early time point of the CLP “cecal ligation and perforation” model (18). 

On the other hand, after 30 days of liver damage, increases in gene expression of *TNF-α, IL-1β, IL-6 *and *TLR4, TGF-β, Foxp3 *and* IL-10* were detected. Preliminarily, it is feasible to speculate that an immune dysfunction induced by the imbalance between pro- and anti-inflammatory molecules had been established by this time point or that translocating bacteria overloaded the regulatory response. However, more specific experiments are needed to elucidate the impact of these cytokines on the permissibility of bacterial translocation, as well as characterize by flow cytometry the differing immune cell populations, which will provide more information and a broader view of the immunological factors at play.

The composition of the intestinal microbiota is important for the maintenance of a symbiotic relationship with the host (19). The bile acids play a crucial role in the composition of the microbiota. Indeed, feeding rats with colic acid has an important impact upon the microbial community in the gut, with a significant increase in the Firmicutes population and a relative reduction in Bacteroidetes (20). However, there are no previous studies, using the BDL model, where the intestinal microbiota of the colon content has been evaluated. The results obtained in the present work show that the obliteration of bile acids in the BDL model induces significant changes in the composition of intestinal microbiota. These changes are characterized by the decrease in the percentage of the relative abundance of Firmicutes and the increase of Gram-negative bacteria such as Bacteroidetes and Proteobacteria. It is worth mentioning that the bacteria belonging to these last two phyla have the peculiarity of adhering to and/or degrading the oligosaccharides of the intestinal mucosa and are related to processes of chronic inflammation (21, 22). On the other hand, the increase in Gram -negative bacteria could be attributed to the fact that in our experimental model the bile flow decreases abruptly, which could favor bacterial proliferation.

The genus *Prevotella*, which is characterized by Gram -negative bacteria responsible for the degradation of fiber from the diet, has been reported to have immunomodulatory properties and contribute to the maintenance of intestinal homeostasis (23). In the 8 days post-BDL group we observed a dramatic decrease (around 11 times) of this genus when compared to the control group; this could have an impact on the production of short chain fatty acids and their regulatory effect on the intestinal barrier.

Similarly, a dramatic decrease in the genus *Lactobacillus* was observed in the colon contents of the groups with liver damage by BDL when compared to the control; at 8 days the decrease was up to 16 times, and at 30 days post-BDL the decrease was approximately 3 fold. These bacteria are distinguished by the production of lactic acid that limits bacterial growth, and have also been described as beneficial for intestinal health because they promote the change of antibody isotype to IgA, by activating EGFR and induction of APRIL (24). Also, in the BDL groups we observed a dramatic decrease in the percentage of other beneficial bacteria that promote host intestinal homeostasis such as* Ruminococcus *(25,26). It is probable that in our experimental model the decrease of these genera may be affecting the bioselectivity of the intestinal microbiota, thus leading to the overgrowth of pathogenic bacteria.

With respect to the above, an interesting fact obtained from this study was the progressive increase of *Sarcina, Clostridium, Turicibacter* and *Helicobacter* in parallel with the liver damage by BDL. Bacteria of the genus *Sarcina* are characterized by proliferation in conditions of gastric outlet obstruction, ulcers or gastric perforation, and it has been reported that this growth has been correlated with an increase in inflammation and damage to the architecture of the intestinal epithelium (27); this point is worth mentioning as it coincides with our experimental model where a delay of gastric emptying has been observed (28). Palm *et al.*, 2014, reported that the family *Erysipelotrichaceae* (family to which *Turicibacter* belongs) is highly immunogenic, and has been associated with pathologies related to chronic inflammation such as colon cancer and Crohn’s disease, as well as HIV infection (29–31). Future studies in our experimental model will primarily focus on transplanting specific microbiota, and determining if they are associated with BT, the evaluation of immunological alternations in the gut and MLN, and the progression of hepatic damage.

**Table 1:** Primer sequences of pro-inflammatory and regulatory molecules

**Gene**	**Sequence**		**Size** **(bp)**	**TM (°C)**	**Reference**
*Rat 2m-F*		CGAGACCGATGTATATGCTTGC	114	60	(9)
*Rat 2m-R*		GTCCAGATGATTCAGAGCTCCA			
*Rat TNF--F*		CACCACGCTCTTCTGTCTACTG	281	60	(10)
*Rat TNF--R*		AGATAAGGTACAGCCCATCTGC			
*Rat IL-1-F*		GGCTTCCTTGTGCAAGTGTC	202	63	NM_031512.2
*Rat IL-1-R*		TGTCGAGATGCTGCTGTGAG			
*Rat IL-6-F*		GCCAGAGTCATTCAGAGCAATA	109	60	NM_012589.2
*Rat IL-6-R*		TTAGGAGAGCATTGGAAGTTGG			
*Rat TLR4-F*		CTCACAACTTCAGTGGCTGGATTTA	177	63	NM_019178.1
*Rat TLR4-R*		GTCTCCACAGCCACCAGATTCTC			
*Rat TGF-1-F*		TCCTTGCCCTCTACAACCAAC	115	63	(11)
*Rat TGF-1-R*		TCCACCTTGGGCTTGCGACC			
*Rat Foxp3-F*		TGAGCTGGCTGCAATTCTGG	115	63	NM_001108250.1
*Rat Foxp3-R*		ATCTAGCTGCTCTGCATGAGGTGA			
*Rat IL-10-F*		TCAAGGAGCATTTGAATTCCC	218	58	NM_012854.2
*Rat IL-10-R*		TTTCATTTTGAGTGTCACGTA			

a) Representative images of liver sections with Masson’s trichrome staining of sham, 8 and 30 days post-BDL and Ishak analysis. b) Biochemical tests: total bilirubin (mg/dl), alkaline phosphatase (IU/l), and ALT (U/l) in sham, 8 and 30 days post-BDL. Sham group represents control rats of 8 and 30 days Results were expressed as mean±SD. Five rats per group. *P-values*≤0.05 were considered as statistically significant. Bars represent 300 μm. BDL: bile duct ligation; ALT: alanine aminotransferase. *(*P≤*0.05); **(*P≤*0.01) and ***(*P≤*0.001)

**Table 2.** Alpha diversity indices of bacterial 16S sequences

**Group**	**Chao1 Ave.**	**Shannon Ave.**
Sham MLN	9.28	2.20
8 days MLN	9.86	2.43
8 days liver	4.66	1.58
Sham colon contents	46.50	3.26
8 days colon contents	36.30	3.11
30 days colon contents	48.65	3.26

MLN: Mesenteric lymph node

a) *TNF-α, TLR4, IL-6 *and* IL-1β* relative gene expression by 2^-DDCT^ analysis. b) *TGF-β, Foxp3* and *IL-10* relative gene expression by 2^-DDCT^ analysis. Beta-2 microglobulin (*β*-2m) was used as a reference gene. Sham group represents control rats of 8 and 30 days. Results were expressed as the mean±SD. Five rats per group. *P-values*≤0.05 were considered as statistically significant. BDL: bile duct ligation. *(*P≤*0.05); **(*P≤*0.01)

## Conclusion

Intestinal microbiome composition is an important factor for the maintenance of a general state of health. During cholestasis, a dysbiotic state in the gut and the mesenteric lymph nodes, is established; in this state bacteria of the Proteobacteria phylum predominate. This, combined with an imbalance in the immune response, can be an important trigger for bacterial translocation and the perpetuation of liver damage. 
